# Diagnostic landscape of first-time cytometric screening for paroxysmal nocturnal hemoglobinuria in Poland in 2013–2022

**DOI:** 10.1186/s13023-024-03283-x

**Published:** 2024-07-17

**Authors:** Justyna Spychalska, Magdalena Duńska, Anna Myślińska, Monika Majewska-Wierzbicka, Edyta Klimczak-Jajor, Eliza Głodkowska-Mrówka

**Affiliations:** 1 Department of Hematological and Transfusion Immunology, Institute of Hematology and Transfusion Medicine, Chocimska 5, Warsaw, 00-791 Poland; 2 Department of Laboratory Diagnostics and Clinical Immunology of Developmental Age, Medical University of Warsaw, Warsaw, Poland

## Abstract

**Background:**

Paroxysmal nocturnal hemoglobinuria (PNH) is an acquired hematopoietic stem cell disorder characterized by PIG-A mutations, leading to glycophosphatidylinositol (GPI)-anchored proteins deficiency that triggers hemolysis – a hallmark of the disease. PNH diagnostics is based on high-sensitivity multicolor flow cytometry (MFC), enabling to detect even small populations of PNH cells. In this single-center, retrospective study, we aimed to characterize a cohort of PNH clone-positive patients first time screened from January 1st, 2013 until December 31st, 2022 with MFC according to International Clinical Cytometry Society PNH Consensus Guidelines.

**Results:**

Out of 2790 first-time screened individuals, the presence of PNH clone in neutrophils was detected in 322 patients, including 49 children and 273 adults. Annual incidence was stable at a median of 31 patients (14 and 19 with clone sizes ≤ 1% and > 1%, respectively), with a decline in number of patients with clone sizes > 1% observed in 2020, potentially influenced by the COVID-19 pandemic. The most common screening indications were aplastic anemia and other cytopenias.

**Conclusions:**

A significant underrepresentation of hemolytic patients was observed as compared to the published cohorts suggesting that these patients are missed in diagnostic process and classic PNH remains underdiagnosed in Poland.

## Introduction

Paroxysmal nocturnal hemoglobinuria (PNH) is an acquired, nonmalignant, clonal hematopoietic stem cell disorder characterized by somatic mutations in PIG-A gene accompanied by additional secondary events leading to defective biosynthesis of cell-surface membrane glycophosphatidylinositol (GPI) [1]. The progeny of the mutated stem cell shows varying degree of deficiency in GPI-anchored proteins, including complement inhibitors, adhesion molecules, blood group antigens, receptors, enzymes, and other functional proteins [2]. Despite lack of intrinsic growth advantage, PNH clone has the ability to expand in patients with underlying immune-mediated bone marrow failure [3]. Deficiency of complement regulatory proteins, such as CD55 and CD59, causes chronic complement-mediated intravascular hemolysis of GPI-deficient red blood cells (RBCs) – a hallmark clinical manifestation of PNH.

Current gold standard for diagnosis of PNH clone involves multicolor flow cytometry (MFC) [4], allowing to rapidly detect GPI-deficient cells based on decreased surface expression of GPI-anchored proteins and/or using fluorescently-labeled proaerolysin (FLAER) that serve as a fluorescent probe specifically binding to the GPI anchors on the cell membrane. High sensitivity assays, widely used since 2013 with subsequent protocol modifications, allow to detect PNH clones with a sensitivity down to 0.01% [5, 6]. As these protocols detect also small populations of PNH cells, introduction of high-sensitivity assays changed our understanding of PNH pathophysiology beyond the hallmark hemolysis and highlighted the significance of subclinical PNH clones in bone marrow failure setting [7]. Here, we present the results of retrospective chart review of a large population of patients screened for the presence of PNH clone over a period of 10 years (2013–2022) using high-sensitivity MFC at a single laboratory in a tertiary hematology center in Poland.

## Materials and methods

### Study design and patient population

We performed a systematic, retrospective chart review covering the records of consecutive patients first time screened for PNH in our laboratory from January 1st, 2013 until December 31, 2022. The patients were referred to our laboratory for high-sensitivity PNH clone screening from a total of 35 hematological centers in Poland. Due to the characteristics of Polish healthcare system, estimation of population coverage of these hospitals is not possible. The choice of cell lineage tested was at referring physician discretion. No additional investigations, in addition to PNH screening from peripheral blood, were undertaken. Clinical data was obtained from referral documentation (external testing, 65.6%) or medical chart review (patients treated and diagnosed in our center, 34.4%). Only patients first-time tested for PNH were included in the analysis.

In total, records of 2790 consecutive patients were analyzed. Of these, the presence of PNH clone in neutrophils was detected in 346 patients, using high-sensitivity MFC. A case was defined to be PNH positive when GPI deficient cells were found in ≥ 2 lineages (neutrophils, monocytes, and/or RBCs) at ≥ 0.02%. As some patients were referred to be tested only in neutrophil lineage, the final analysis was based on 322 patients including 49 children (median age 12) and 273 adults (median age 42) (Table 1), for whom data of at least neutrophil and a single additional lineage was available.

**Table 1:** Characteristics of the study group

Demographics						
	Sex, n, %		Age, years			
	Male	Female	Median	95% CI	Mean	Range
All	176 (54.7%)	146 (45.3%)	36	32–40	40	2–85
Children	28 (57.1%)	21 (42.9%)	12	11–14	12	2–17
Adults	148 (54.2%)	125 (45.8%)	42	37–47	45	18–85
Median clone size in respective lineages, %						
	Neutrophils		Monocytes		Erytrocytes (total)	
	Median	95% CI	Median	95% CI	Median	95% CI
All	1.95	1.28–3.25	3.2	1.89–5.3	0.30	0.20–0.45
Children	1.72	0.40–4.32	5.30	1.11–7.95	0.17	0.07–0.50
Adults	1.97	1.28–3.50	3.12	2.15–5.57	0.34	0.21–0.51
Indications for screening (n, %)						
	AA	Hypoplasia/cytopenia	MDS	Hemolytic	Thrombotic	Other
All	141 (43.8%)	105 (32.6%)	40 (12.4%)	27 (8.4%)	4 (1.2%)	5 (1.5%)
Children	36 (73.5%)	11 (22.4%)	-	1 (2.0%)	-	1 (2.0%)
Adults	105 (38.5%)	94 (34.4%)	40 (14.6%)	26 (9.5%)	4 (1.5%)	4 (1.5%)

*Abbreviations*: *95% CI* 95% confidence interval, *AA* Aplastic anemia, *MDS* Myelodysplastic syndrome

### Ethics

The study was performed in accordance with the Declaration of Helsinki ensuring data confidentiality. The study protocol was approved by the local IHTM Bioethics Committee (26/2023) with a waiver of the documentation of informed consent.

### Flow cytometry

**Fig. 1:**
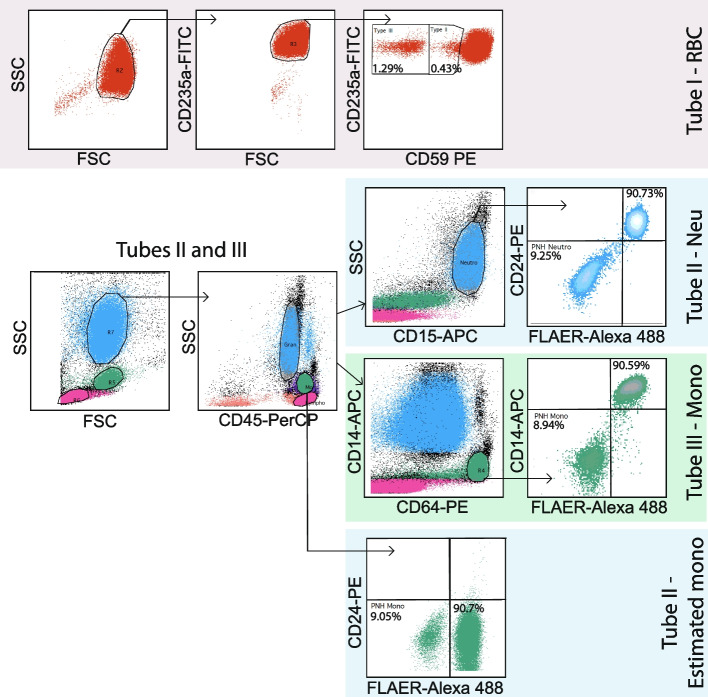
Gating strategy for paroxysmal nocturnal hemoglobinuria flow cytometry assay. A normal whole blood sample was stained as described in Materials and Methods and Table 2. The representative dot plots from a single PNH-positive patient are shown. High-sensitivity assay is shown in the upper panels (tube I for RBC, tube II for neutrophils, and tube III for monocytes), while estimation of monocyte PNH-clone is shown in the lowest panel (from tube II)

Flow cytometry protocol was established based on International Clinical Cytometry Society (ICCS) Consensus Guidelines [4, 8] and further validated to be used in our laboratory. Peripheral blood samples (EDTA) were tested within 48 h from collecting. In tube I: RBCs were analyzed with 2-color reagent cocktail, i.e. CD235a/CD59 [6]. Leukocytes were analyzed, using 2-tubes method with 4-color reagent cocktail, i.e.: tube II: FLAER/CD24/ CD15 for neutrophils [4, 8], and tube IIIa: FLAER/CD14/CD45/CD33 [9] (January 2013 – April 2016) or tube IIIb: FLAER/CD14/CD45/CD64 [4, 8] (May 2016 – December 2022) for monocytes. Detailed information on specific clones and conjugates as well as gating strategy are presented in Table 2 and Fig. 1, respectively. In cases where high-sensitivity test for monocytes was not ordered by the referring physician (the screening was performed in RBCs and neutrophils only), the clone size in monocytic lineage was retrospectively estimated based on the results of neutrophil staining (CD45^pos^ CD24^neg^ population, Fig. 1). Samples acquisition and analysis were performed on BD FACSCalibur™ cytometer with BD CellQuest Pro™ software. Acquisition for each high sensitivity test was up to 250 000 cells. Limit of detection (LOD, minimum 20 GPI-deficient cells) was down to 0.01%, lower limit of quantification (LLOQ, minimum 50 GPI-deficient cells) was down to 0.02% for neutrophils and RBCs, and 0.04% for monocytes. PNH clone was expressed as a sum of type II cells (partial GPI deficiency) and type III cells (total loss of GPI expression) [4]. If not indicated otherwise, the clone size provided refers to the neutrophil lineage.

**Table 2:** Specification of antibodies used for PNH screening

Tube I				
RBC	Ab	Fluorochrome	Clone	Manufacturer
	CD235a	FITC	YTH 89.1	invitrogen
	CD59	PE	MEM-43	invitrogen
Tube II				
NEU	Ab	Fluorochrome	Clone	Manufacturer
	CD45	PerCP	2D1	BD Biosciences
	CD15	APC	HI98	BD Pharmingen
	FLAER	Alexa Fluor 488	N/A	Cedarlane
	CD24	PE	ALB9	Beckman Coulter
Tube IIIa				
MONO	Ab	Fluorochrome	Clone	Manufacturer
	CD45	PerCP	2D1	BD Biosciences
	CD33	PE	P67.6	BD Biosciences
	FLAER	Alexa Fluor 488	N/A	Cedarlane
	CD14	APC	MФP9	BD Biosciences
Tube IIIb				
MONO	Ab	Fluorochrome	Clone	Manufacturer
	CD45	PerCP	2D1	BD Biosciences
	CD64	PE	10.1	BD Pharmingen
	FLAER	Alexa Fluor 488	N/A	Cedarlane
	CD14	APC	MФP9	BD Biosciences

*Abreviations*: *Ab* Antibody, *RBC* Red blood cells, *NEU* Neutrophils, *MONO* Monocytes

### Statistical analysis

Normality of data distribution was evaluated using Shapiro–Wilk test. Data comparisons were performed using mixed-effect ANOVA (for quantitative values with missing values) or two-way ANOVA (for quantitative values without missing values). The relationships between the observed values were evaluated using Spearman's rank correlation coefficient. A *P* value below 0.05 was considered significant. Statistical analysis, descriptive statistics, and data visualization were performed using GraphPad Prism 10 version 10.0.2 for Windows, GraphPad Software, Boston, MA.

## Results

**Fig. 2:**
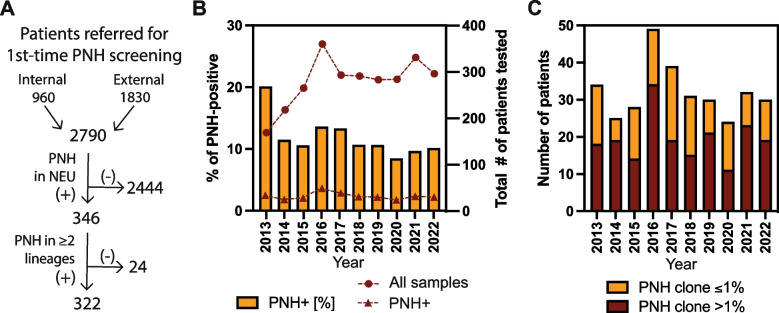
Annual frequency of PNH cells/ clone in the studied population in 2013–2022. **A** Flow diagram representing inclusion and exclusion criteria in the studied population. **B** The number of first-time referrals for PNH screening (dots) and cases that tested positive for the presence of PNH clone in any size in at least two lineages (absolute numbers – triangles, percentages – bars) in the studied period. **C** The number of patients with PNH clone in neutrophil lineage at ≤ 1% (yellow bars) and > 1% (maroon bars) in the studied period

Out of 2790 patients examined in our laboratory as a part of PNH screening, a total of 322 cases (11.5%) with GPI-deficient cells in ≥ 2 lineages (neutrophils, monocytes, and/or RBCs) at ≥ 0.02% were identified and included for further analysis (Fig. 2A). The clone size with high sensitivity assay in neutrophil lineage was evaluated in all cases, in erythrocyte lineage in 309/322 cases, and in monocytic lineage in 34/322 cases. In 239/322 cases clone size in monocytic lineage was estimated based on the results of neutrophil staining in tube II (CD45^pos^ CD24^neg^ cells, Fig. 1). All three lineages were tested in 21/322 cases. The number of patients referred to our laboratory per year was growing gradually during the first 4 years after introduction of high sensitivity protocol, and after 2016 remained stable with a median of 288 (95% CI 218–331) (Fig. 2B). In the studied period, annually a median of 31 patients with PNH clone was diagnosed (95% CI 25–39), including a median of 14 patients with rare PNH cells (clone size 0.02–0.1%) or very small clones (0.1–1%) and a median of 19 patients with larger clone sizes (> 1%) (95% CI 9–16 and 14–23, respectively). The percentage of patients who tested positive for PNH clone was the highest at the beginning of high-sensitivity screening (20.1%) and stabilized from the second year of testing to achieve a median level of 10.6% (95% CI 9.7–13.6%) (Fig. 2BC). For the entire studied period the observed frequency of PNH clone remained within the confidence interval except for 2020, when markedly lower number of PNH clones was diagnosed (8.45% of positive tests, 24 cases including 11 with clone size > 1% in 2020 in comparison to a median of 10.6%, 31 and 19, respectively for 2013–2022) (Fig. 2BC).

**Fig. 3:**
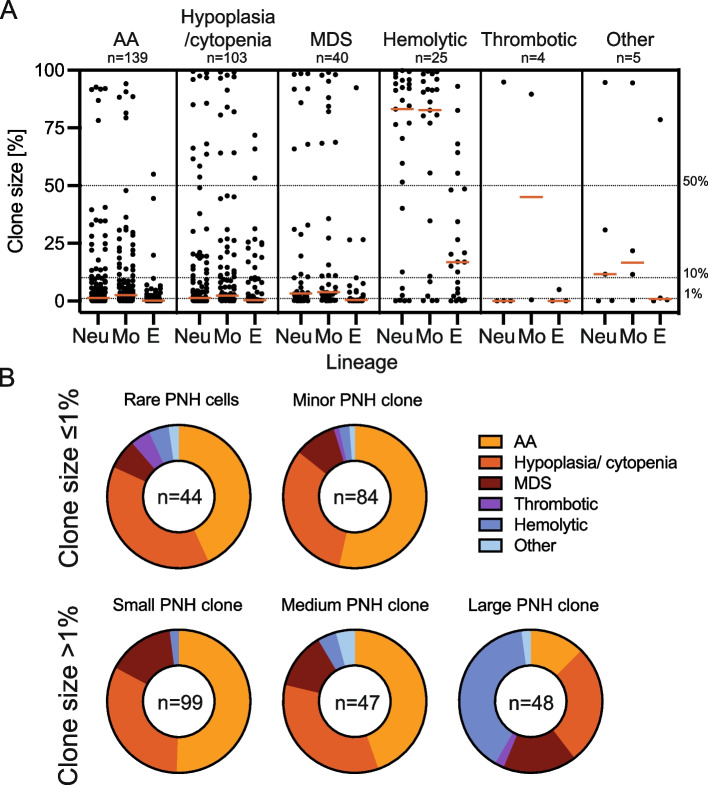
Indications for PNH screening and respective clone sizes. **A** Scatter plot representing clone size in neutrophil (Neu), monocyte (Mo) and total type II and type III RBC (E) lineages of all individual patients depending on primary diagnosis. Each dot represents a single patient data. Red horizontal line represents median values. Dotted lines show the levels of 1%, 10%, and 50% of PNH clone. “Other” include secondary PNH to hematopoietic stem cell transplant (*n* = 1) and no data (*n* = 4). **B** Indications for screening in populations with different clone sizes shown as a % of patients in each group. Clone sizes were defined as follows: rare PNH cells – 0.02–0.1% of GPI-deficient cells in neutrophil lineage, very small PNH clone – 0.1–1%, small PNH clone – 1–10%, medium PNH clone – 10–50%, and large PNH clone 50–100%

The most common underlying condition that prompted PNH screening were aplastic anemia (AA) and other cytopenias and/or bone marrow hypoplasia (*n* = 141, 43.8% and *n* = 105, 32.6%, respectively), followed by MDS (*n* = 40, 12.4%). All but 3 pediatric cases of PNH clone (*n* = 46, 93.9%) were referred due to cytopenia or AA. Two pediatric patients were referred due to hemolytic anemia with normal bone marrow function, and a single patient due to unknown reason (Table 1). In adult patients, hemolytic or thrombotic presentations with normal bone marrow were much less commonly observed than bone marrow failure (*n* = 27, 8.4% and *n* = 4, 1.2%, respectively) (Fig. 3A). In general, median clone size in patients with bone marrow failure syndromes (AA, hypoplasia/ cytopenia, MDS) was lower than in patients with hemolytic presentation of PNH. In a small population of patients with thrombotic presentation, 3 out of 4 (75%) patients had very small PNH cell population (rare PNH cells defined as clone size 0.02–0.1% and minor PNH clone defined as clone size of 0.1–1%) in all lineages tested, and only 1 (25%) had a large clone (defined as > 50% clone size) in neutrophil lineage (Fig. 3A). Almost all patients with very small clone sizes were diagnosed with bone marrow failure syndromes (AA, hypoplasia/ cytopenia or MDS), except for 7 patients with normal bone marrow and hemolytic (*n* = 4, 3.1%) or thrombotic (*n* = 3, 2.3%) presentation and rare PNH cells detected in MFC (Fig. 3B). Most patients with classical PNH (hemolytic presentation without bone marrow failure) had large clones in neutrophil and/or monocytic lineages (*n* = 19, 70.3% of all patients with hemolytic presentation and normal bone marrow), however, due to predominance of patients with bone marrow failure syndromes in our cohort, most patients with large clones had underlying bone marrow failure (*n* = 28, 57.1%) (Fig. 3B).

**Fig. 4:**
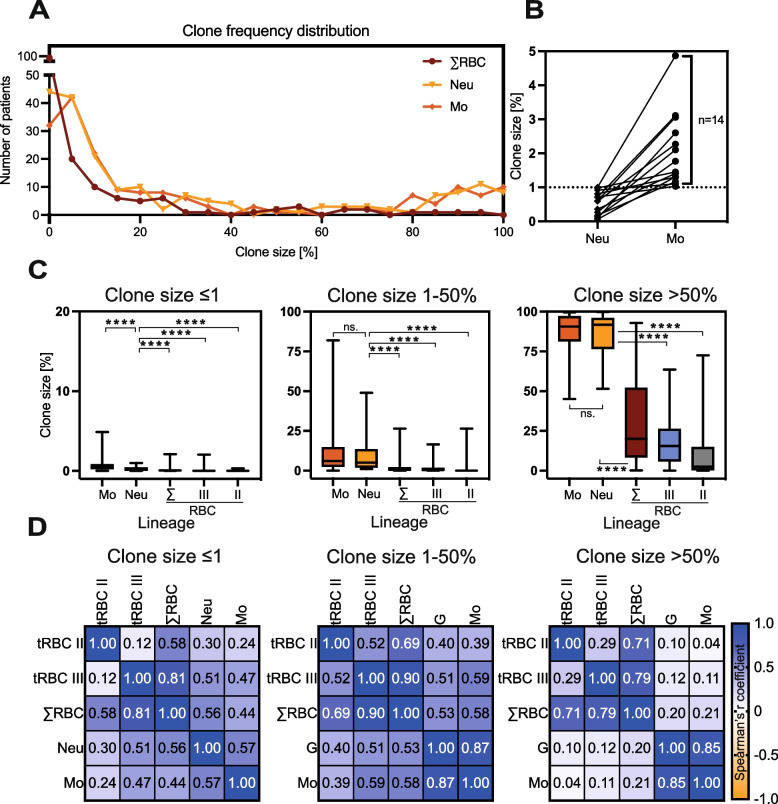
Distribution of clone size and correlation of clone sizes in different lineages in the studied population. **A** Clone size frequency distribution depending on cell lineage (neutrophil – N, monocyte – Mo, and total type II and type III RBC – ΣE) in patients with neutrophil clone > 1%. Each data point (dots for RBCs, triangles for neutrophils, and diamonds for monocytes) of the histogram represents total number of patients (Y axis) grouped in “bins” of 5% clone size width each (X axis). **B** Clone size (%) in neutrophil (N) and monocyte (Mo) lineages in a group of patients with neutrophil clone ≤ 1% highlighting *n* = 14 patients (14.4%) with monocyte clone exceeding 1%; dots represent clone size in the respective lineage, lines connect clone sizes of the same patients, *P* value (**C**) Box and whiskers plot of PNH clone sizes in three peripheral blood cell lineages (neutrophil, monocyte, and RBC) in a population of *n* = 128 patients with very small PNH clone (≤ 1%, left panel), *n* = 146 patients with small to medium PNH clone (1–50%, middle panel), and *n* = 48 patients with large PNH clone (> 50%, right panel). Median value (horizontal line) together with 25th percentile ranges (box) and range (whiskers) are shown. **D** Spearman’s r correlation matrix for leukocyte and red blood cell PNH clone size in a population of *n* = 128 patients with very small PNH clone (≤ 1%, left panel), *n* = 146 patients with small to medium PNH clone (1–50%, middle panel), and *n* = 48 patients with large PNH clone (> 50%, right panel). Note: If not indicated otherwise, the clone size provided refers to the neutrophil lineage. Abbreviations: AA – aplastic anemia, E and ΣE – total RBCs (type II and III), E II – type II RBCs, E III – type III RBCs, MDS – myelodysplastic syndrome, Mo – monocytes, Neu – neutrophils

Distribution of PNH clone sizes was bimodal for neutrophil and monocyte lineages, however, in RBC lineage it was unimodal lacking peak at large clone sizes (Fig. 4A). Median clone size was uniformly larger in monocytic lineage in comparison to neutrophil lineage (3.20% vs 1.95%, *p* < 0.001) (Fig. 4B, C). Importantly, in 14.9% (14/94) patients with ≤ 1% clone in neutrophils for whom data on monocytes was available, PNH clone in monocytic lineage exceeded 1% (Fig. 4B). Due to the sensitivity of RBCs to complement-mediated hemolysis and the interference of transfused RBCs with clone size evaluation, median clone sizes in RBCs were significantly smaller, especially in patients with hemolysis and/or after transfusion (Table 1, Figs. 3A and 4). The analysis of the relationship between clone sizes in the analyzed populations showed strong positive correlation for neutrophil and monocyte lineages for clones 1–50% (Spearman’s rank correlation coefficient *r* = 0.82) and moderate positive correlation for other clone sizes (*r* = 0.59 for clones < 1% and 0.76 for clones > 50%) (Fig. 4D). The correlations between leukocyte lineages (Neu, Mo) and RBCs were much weaker for clones < 50% or practically negligible for large clones (Fig. 4D).

## Discussion

As is often the case of many other rare diseases, PNH incidence has been poorly reported with just a few published registry studies, none of them covering Polish population. Based on the available data, PNH incidence is generally estimated at 1–1.8 cases per million individuals worldwide uniformly affecting population with different genetic backgrounds [2, 10, 11]. Extrapolating these values, in Polish population of 37.75 million, a range of 38–68 new cases could be expected annually. In the studied period, a total of 322 patients with a median of 31 cases a year with PNH clone of any size (95% CI 25–39) and 19 cases a year with PNH clone > 1% (95% CI 14–23) were diagnosed in our laboratory, that theoretically comprise 45.6–81% of the predicted cases. Despite the single-center nature of our data, that comprise major limitation of the study, we believe that this dataset can be considered representative for Polish population.

The annual number of diagnosed cases did not differ significantly within the studied period and remained at the stable level with a single outlying value in 2020, when COVID-19 pandemic exerted the most pronounced influence on healthcare systems worldwide. In that period, although the number of referred patients did not differ from median for the studied period (*n* = 284 vs median of 288, 95% CI 218–331), the number of those who tested positive for PNH clone (*n* = 24 vs median of 31, 95% CI 25–39, and 8.45% vs median of 10.63%, 95% CI 9.67–13.61) and the number of patients with PNH clone > 1% (*n* = 11 vs median of 19, 95% CI 14–23, and 3.9% vs median of 6.7, 95% CI 5.1–9.4) were significantly lower than the observed median (Fig. 2BC). Numerous studies have confirmed that due to pandemics, the access to healthcare services in 2020, including care provided by hematology centers in Poland, was markedly limited, resulting in decreased number of hospitalizations and delayed diagnoses made in the affected period [12, 13]. Contrary to this trend, lower PNH clone detection rate in total and more frequent detection of small clones observed in our cohort, did not result from decreased number of referrals, as this value remained at the expected level. Recent studies have shown that the incidence of idiopathic AA continuously declined from the beginning of the pandemics until the end of 2020 [14] that may explain decreased PNH clone detection rate in our cohort, especially that AA patients constituted the majority of the PNH-positive patients in the study group. The changes in hematology practice in the country or diagnostic access bias (preferential access to services for cytopenic and MDS patients with small clones) cannot be excluded. Detailed analysis of this phenomenon is beyond the scope of this study; however, it might be speculated, that the reduced PNH clone detection rate might be caused by factors directly or indirectly related to COVID pandemics.

In our cohort, 322/2790 (11.5%) new cases of PNH clone were detected, including 40.6% of cases (128/322) with very small clone or rare PNH cells (≤ 1%) (Fig. 2BC). Similar detection rate was observed in Spain (10%) [15], while other centers reported much higher (16% in Brazil [15]) or lower (6.48% in Dahl‐Chase Dx Services, Bangor, US [8]) rates that may be related to local clinical practice and varying indications for screening. Similarly to our observations, very small clones, detected with high-sensitive protocols, were reported for 44% cases in the US cohort [8] confirming the benefits of high-sensitive testing in the PNH screening, especially in bone marrow failure patients who tend to have lower clone sizes.

The most common indication for PNH screening in the published studies are AA and other cytopenias, constituting 34.5%-65.6% of cases, followed by hemolytic anemia as a second commonest indication observed in 19%-30.7% of cases [15–17]. In our cohort, AA and cytopenias were much more common, constituting 76% of cases, while hemolysis was relatively rare indication for screening constituting 8% of cases (Fig. 3). This data suggests a significant underrepresentation of patients with hemolytic anemia in the population screened for PNH clone in routine clinical practice in Poland resulting in relative overrepresentation of AA and cytopenia patients. In our experience, bone marrow failure patients are treated in specialized hematology centers that strictly follow the current guidelines and refer most of these patients to PNH screening [7]. Hemolytic anemia patients, on the other hand, are more often treated outside of tertiary reference hematology centers, and therefore may be not referred for PNH screening as a part of differential diagnosis. Pure thrombotic presentation of PNH was also less common in our cohort in comparison to published data (1.2% vs 2.1% or 22.1% in UK [18] and Belgian [17] cohorts, respectively); however, the differences between reported cohorts are too large to draw any conclusions.

According to the expert consensus on PNH diagnosis, at least 2 cell lineages need to be tested to confirm PNH diagnosis [4]. However, as highlighted in the same guidelines, evaluation of the PNH clone in all three lineages is becoming the standard of care for PNH-suspected cases. As the presented dataset has been collected in real-life clinical setting for a decade, and most of the material has been studied based on previous diagnostic guidelines, most of the patients evaluated in his paper has been tested in two lineages only.

High-sensitivity quality of the assay has been assured by stringent verification and validation of the assay according to the standards set up by the consensus guidelines [4, 8]. As this is a real-life, clinical laboratory dataset, no additional antibody specificities nor additional staining, such as live/dead counterstaining, have been added to the antibody cocktails. Although some authors suggest that the addition of 7AAD or DRAQ7 counterstaining may improve the quality of the data obtained and reduce non-specific reagent binding positively influencing the evaluation of the PNH clone size [19, 20], we decided not to modify the established staining methods described in the current guidelines.

As observed in previous studies [21, 22], in most patients clone sizes in neutrophil and monocyte lineages are closely correlated (Fig. 4D). Interestingly, in a few cases in our cohort with rare PNH cells or very small clone size in neutrophils (*n* = 14, 14.9% of patients with both clones measured), clone sizes exceeding 1% were observed in monocyte lineage highlighting the need to evaluate both leukocyte lineages to achieve comprehensive clone size evaluation (Fig. 4B). Also, median clone size was uniformly higher for monocytes than any other cell lineage tested in all subpopulations (Fig. 4C). We cannot exclude, that this phenomenon may have occur due to different sensitivity of neutrophils and monocytes to cell death, as cell death markers counterstaining was not used [19, 20]. However, in our hands and as described in the current guidelines [4, 8], the sensitivity of PNH clone detection is high even in the absence of counterstaining. Moreover, the difference between monocyte and neutrophil clones was visible regardless of the origin of the sample (samples from our center tested a few hours after blood drawing and samples from other centers tested with a delay allowing for transport).

As expected, clone size of PNH RBCs, due to RBC susceptibility to complement-mediated hemolysis and clone size changes due to blood transfusions, correlate weakly or do not correlate with the size of PNH clones in leukocyte lineages, especially in cases with large RBC clones (Fig. 4D). For the same reason, typical bimodal distribution of clone size [3] was observed for leukocyte lineages, but not for RBCs (Fig. 4A). Noteworthy, the peak for the largest clones in the histogram (Fig. 4A) was lower in comparison to the data published so far confirming underrepresentation of hemolytic patients in our cohort [3, 21].

The major limitations of our study were single-center nature and lack of extensive clinical data. However, although the presented data has been collected in a single laboratory, the studied patients were referred for testing from 35 hematology centers from the entire country that extends the geographical range of the studied population much beyond our region, making the obtained data representative for the country population. On the other hand, the fact that the studies were performed in a single unique cytometry unit offers additional advantage of better standardization of the results obtained. Since a significant number of patients in our cohort were referred to our laboratory for testing only, in these cases data on indication for screening was obtained from referral documents only without chart review, that may have affected data completeness (e.g. due to underreporting of thrombosis or hemolysis in bone marrow failure patients). In this line, as the data on blood transfusion was not available for all patients, we decided not to include this confounding factor that may strongly affect PNH clone size in RBC. In addition, although only patients who tested positive for the presence of PNH cells in two lineages, the choice of the lineage tested was at referring physician discretion. In consequence, data for monocytes and RBCs are not available for all patients that may limit statistical power of some evaluations.

To conclude, we present the largest cohort of patients diagnosed with PNH clones/ cells in real-life routine clinical practice in Poland published so far. Our data confirms the efficiency of high-sensitivity testing recommended by International Clinical Cytometry Society Consensus Guidelines, especially in patients with bone marrow failure. Although our findings are mostly consistent with the available body of data from other countries, we observed marked underrepresentation of hemolytic patients in PNH screened population and PNH-positive cases. Contrary to the expectations, classic PNH was one of the rarest indications for PNH screening, suggesting that in routine practice in Poland differential diagnosis of hemolytic anemia rarely include PNH. This highlights the need for improved education of clinical community about PNH and indications for PNH clone screening. To better understand epidemiology of PNH and patients’ characteristics, initiation of national registry of patients burdened with PNH clone is warranted.

## Data Availability

The anonymized datasets used and analysed during the current study are available at https://libusza.ihit.waw.pl/Datasets/PNH_Dataset.xlsx
